# Supplementary material for: The sociospatial factors of death: Analyzing effects of geospatially-distributed variables in a Bayesian mortality model for Hong Kong
Source: PLoS One. 2021 Mar 24;16(3):e0247795. doi: 10.1371/journal.pone.0247795 (PMC7990297; doi:10.1371/journal.pone.0247795)

S1 Data variables

S1 Fig . Cross-sectional attributes for calendar year 2016 by district including **(A)** mortality rates, **(B)** number of life insurance policies, **(C)** unemployment rates, **(D)** number of homeless individuals, and **(E)** number of mobile residents. The dashed red lines indicate the average value for each of these variables in Hong Kong. The four attributes presented here are not strongly correlated with mortality.

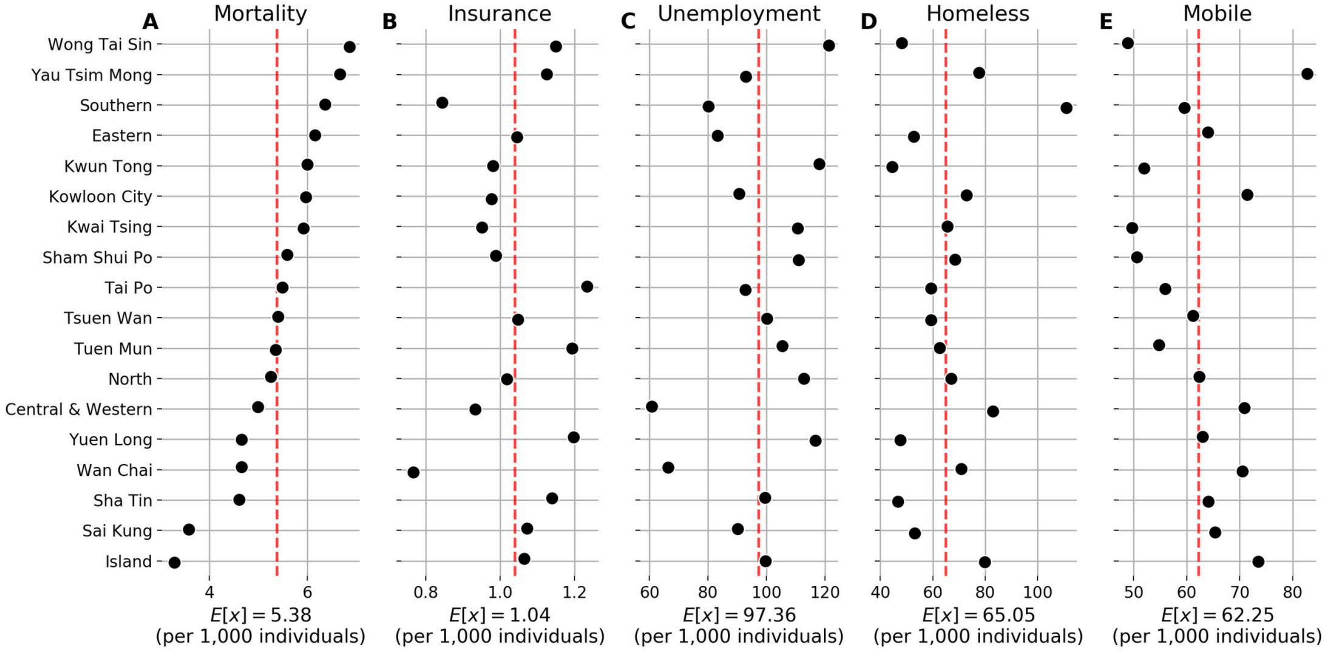

S2 Fig. Households statistics by year.

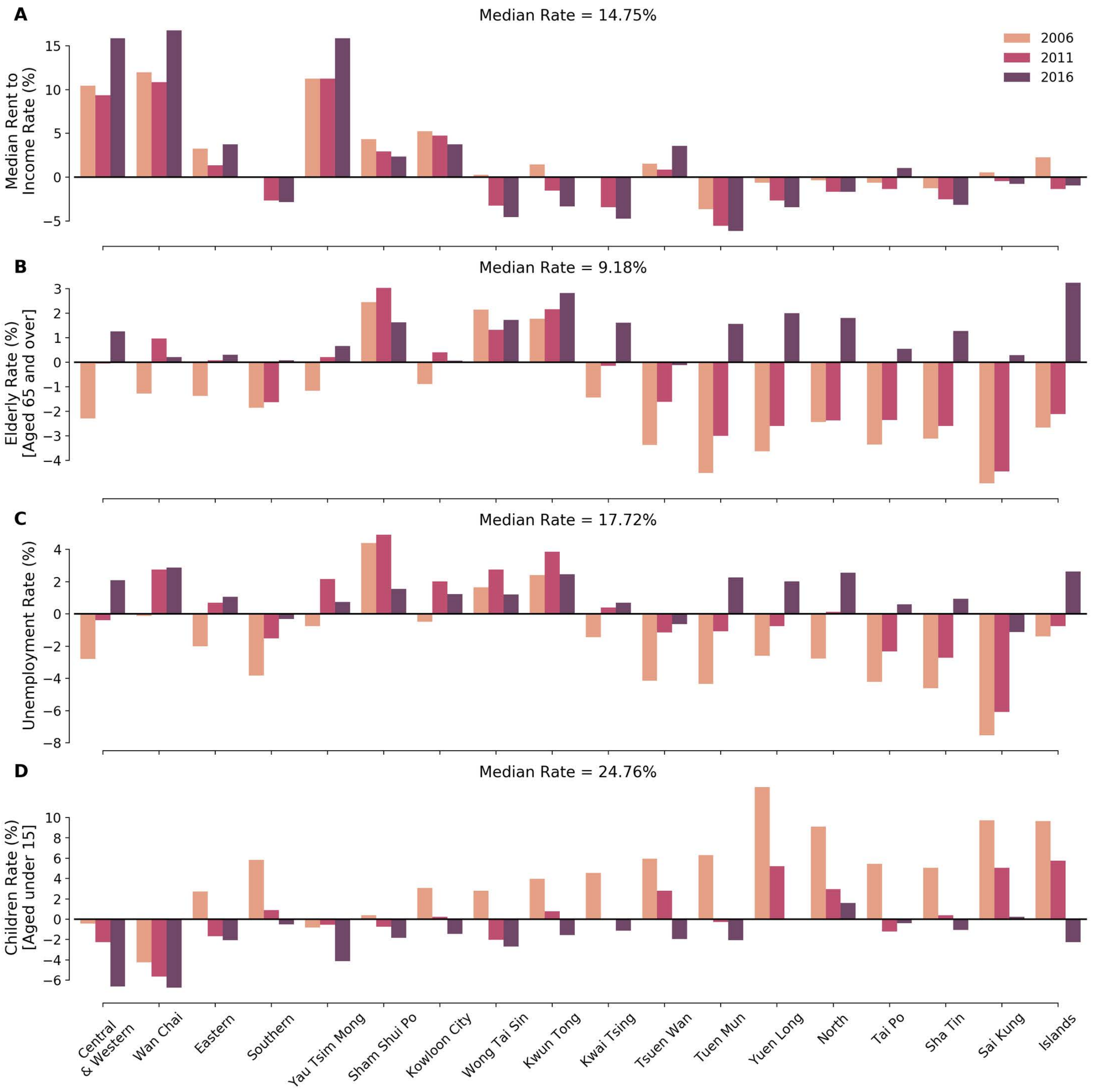

- **Mortality rates**: Annual death rates by districts provided by the Census and Statistics Department of Hong Kong [47]. We derive an annual crude death rate of each district by simply dividing the number of registered death records by the population size for each calendar year such that:

$$Y_{it} = \frac{N_{it}(\text{deaths})}{N_{it}(\text{census population})},$$

for district i and $t \in [2006, 2011, 2016]$.

- **MMA life insurance coverage**: Annual proportion of insurance policies issued in each district and normalized by population size.

- **Median income**: Estimation of median income by the Census and Statistics Department of Hong Kong for each district.
- **Median rent to income ratio**: The percentage of monthly household income spent on monthly household rent in inflation-adjusted terms.
- **Unemployment rates**: The proportion of people without an active job normalized by population size. This is a proxy variable derived from the gap between the available labour force and economically active individuals—aged over 15 and has been working for at least a week in the reference year [46].
- **Proportion of children**: The proportion of young children aged under 15 for each district.
- **Proportion of elderly**: The proportion of seniors aged 65 or above for each district.
- **Median monthly household income**: Similar to median income, but this is computed for households rather than individuals.
- **Unemployment rates across households**: Similar to unemployment rates but derived at the households level rather than individuals.
- **Unemployment rates among minorities**: Similar to unemployment rates but computed for minorities only.
- **Proportion of homeless people**: Another proxy variable to estimate the proportion of people not living in registered domestic households.
- **Proportion of homeless mobile residents**: Hong Kong's permanent residents who had stayed in a given district for "at least 1 month but less than 3 months" during the the 6 months before or after the reference moment [46].
- **Proportion of single parents**: "Mothers or fathers who are never married, widowed, divorced or separated, with child(ren) aged under 18 living with them in the same household" [46].
- **Proportion of households with children in school**: The proportion of households with young adults attending full-time courses in educational institutions in Hong Kong.

S3 Fig. Records by TPU. (A) shows the fraction of records reported by each TPU in the mortality dataset. **(B)** shows an exponential declines in the number of records by TPU whereby the horizontal axis shows TPUs ranked by the fraction of death records reported on the vertical axis. Similarly, **(C)** shows the annual average of deaths for each decade by TPU.

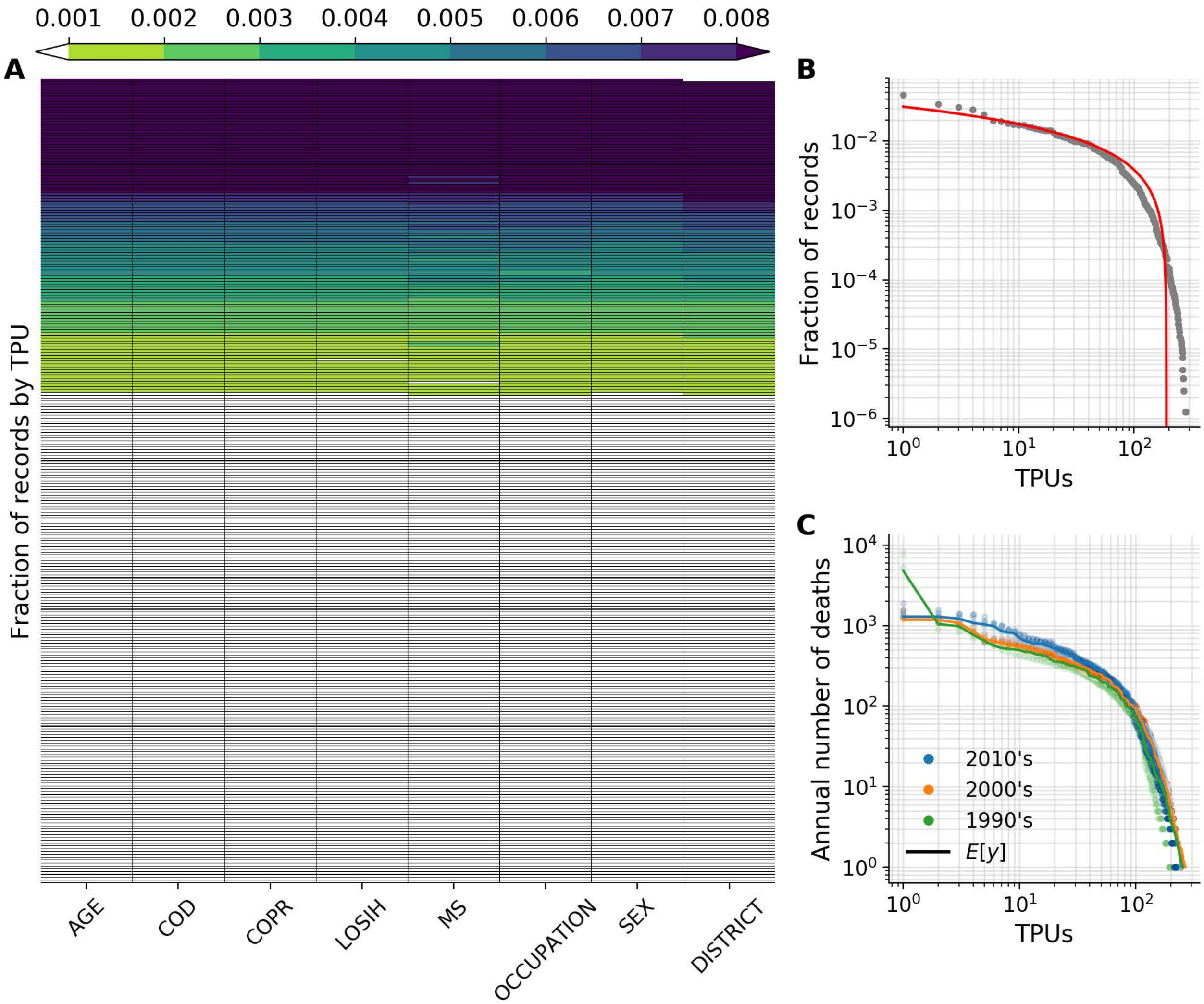

Our initial analysis shows variations in mortality rates across districts. In S1 Fig, we show a cross-sectional analysis of 4 different socioeconomic indices with respect to our response variable (mortality rate) for 2016. We normalize these values to show the number of people in each bin per 1,000 individuals for each variable. The dashed red lines show the average value. Districts with a lower number of mobile residents have higher rates of morality, such as Wong Tai Sin, Kwun Tong, Kwai Tsing, and Sham Shui Po. We note high mortality rates in the Yau Tsim Mong and Southern districts, while having a higher number of homeless people. Many districts with a lower number of homeless people have higher rates of mortality, such as Wong Tai Sin, Eastern, and Kwun Tong, and Kowloon City.

S2 Tertiary Planning Unit (TPU)

A Tertiary Planning Unit (TPU) [34]—similar to a census-block in the US—is a geospatial reference system used by the Hong Kong's Census and Statistics Department to report population census statistics. To inspect the geospatial tags we have in our dataset, we show the fraction of death records found in our data set by each TPU (S3 Fig) and by districts (S4 Fig). Unfortunately, most death records were found in a small fraction of TPUs, which is understandable because of known data privacy concerns about the dataset, as records in small TPUs may reveal sensitive information about specific individuals there. In S5 Fig, we show the annual number of reported deaths by TPU. We note that death records are not reported frequently at the TPU level in the dataset. Missing records are flagged with XXX as stated in the data set dictionary provided by the Hong Kong's Census and Statistics Department.

To avoid any risk of identifying individuals in the dataset, we use districts as our main spatial unit, trying our best to stay away from the pitfalls of data privacy in census data. In fact, most studies have either filtered out small TPUs in their analyses [13, 28], or aggregated their records at the district level [33] to overcome this challenge in the dataset. In S4 Fig, the main 18 districts of Hong Kong show very

S4 Fig. Records by district. (A) shows the fraction of records reported by each district in the mortality dataset. **(B)** shows an exponential declines in the number of records by district whereby the horizontal axis shows districts ranked by the fraction of death records reported on the vertical axis. Similarly, **(C)** shows the annual average of deaths for each decade by district.

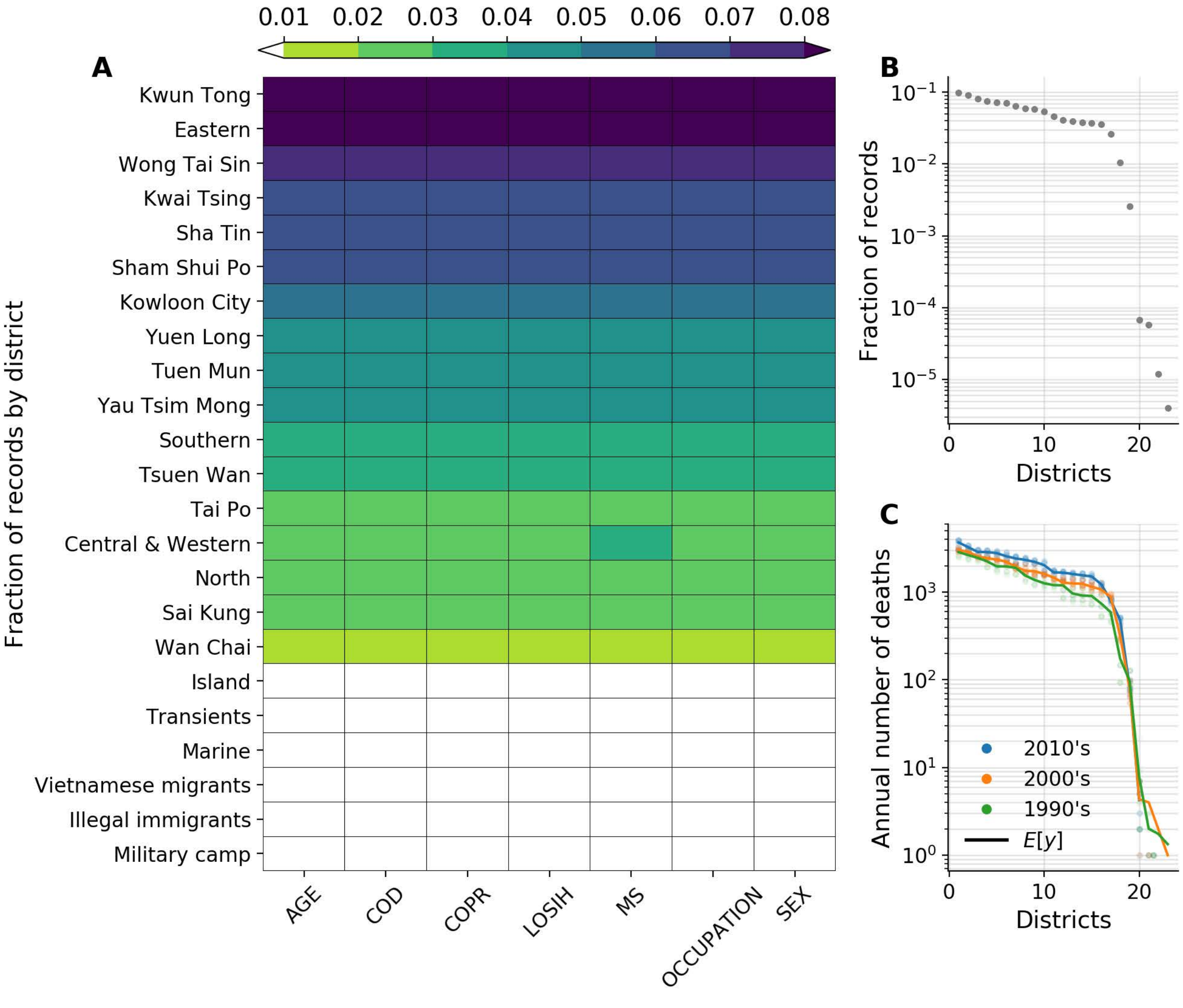

consistent data distributions for all variables in the data set throughout the last few decades. We note that the last 5 entries in the heatmap are not districts per se, but they are only used by the census department to report deaths outside the borders of the main 18 districts. However, given access to a more granular geospatial resolution (*i.e.,* TPU) our method can be implemented in a similar manner to identify and explore some of these sociotechnical dynamics.

S5 Fig. Annual number of deaths by TPU. Death records are not reported frequently at the TPU level in our mortality dataset. We note that the code (XXX) is used to indicate missing labels.

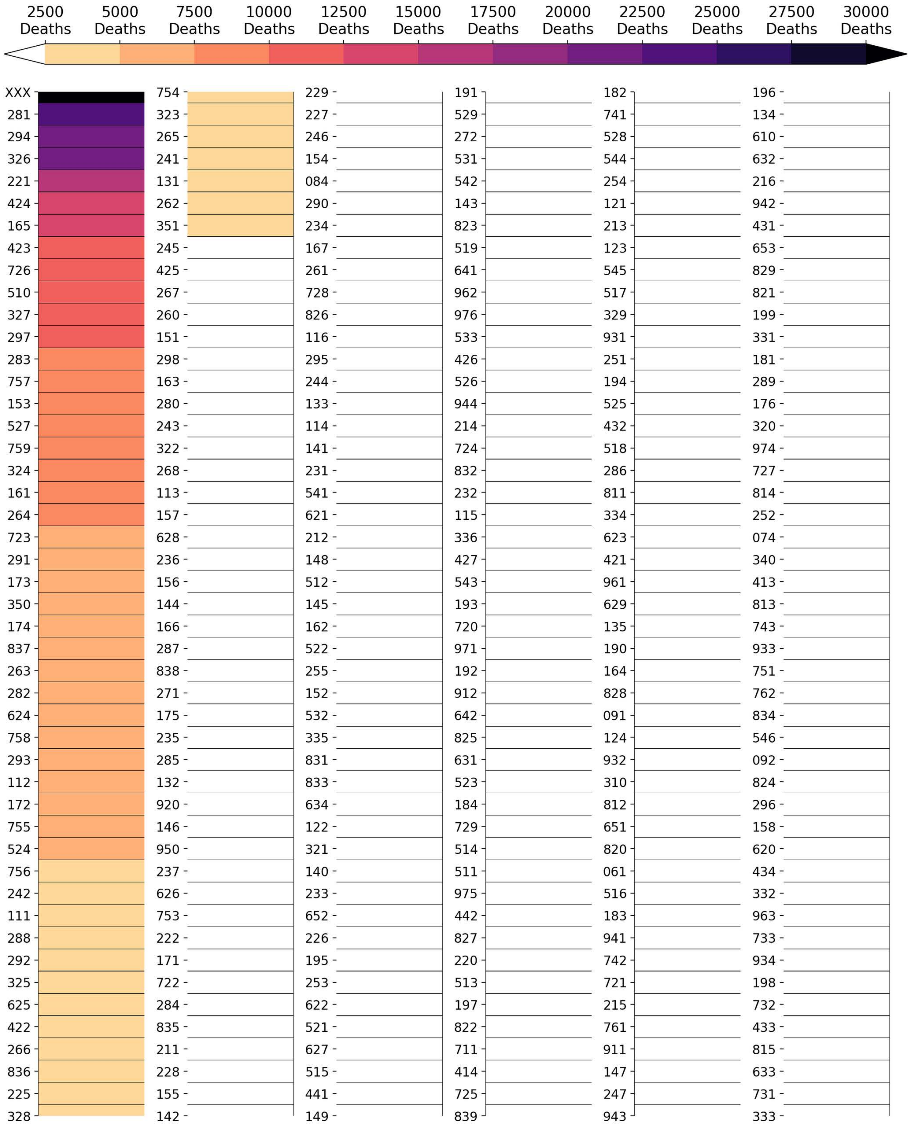

S3 Supplementary Figures

S6 Fig. Distributions of β for the baseline model. We show the kernel density estimation of β coefficient for each feature in the design tensor. Distributions that are significantly above the mean are colored in orange while distributions significantly below the mean are colored in blue as measured by the 80% CI.

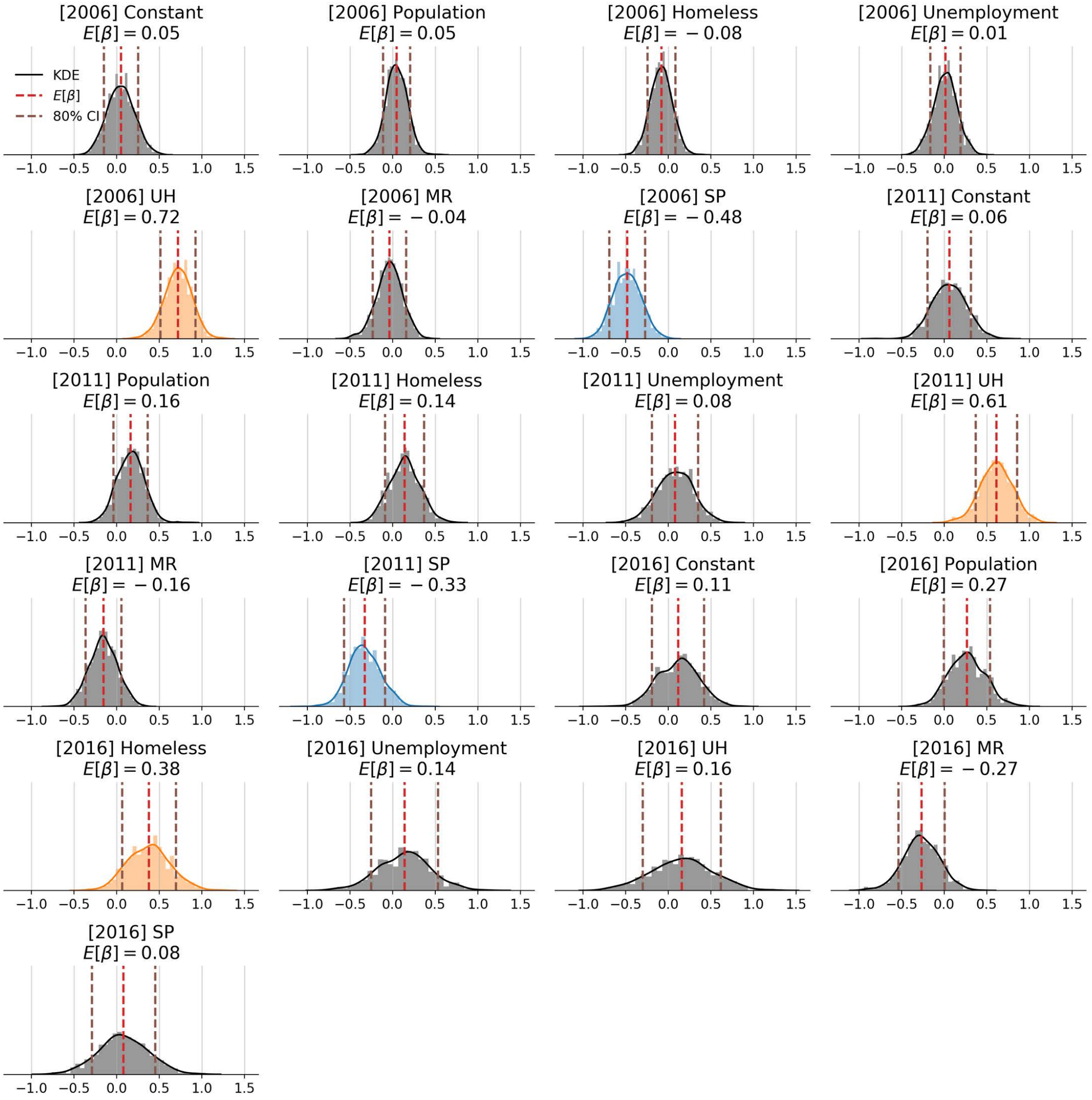

S7 Fig. Distributions of β for the spatial model. We show the kernel density estimation of β coefficient for each feature in the design tensor. Distributions that are significantly above the mean are colored in orange while distributions significantly below the mean are colored in blue as measured by the 80% CI.

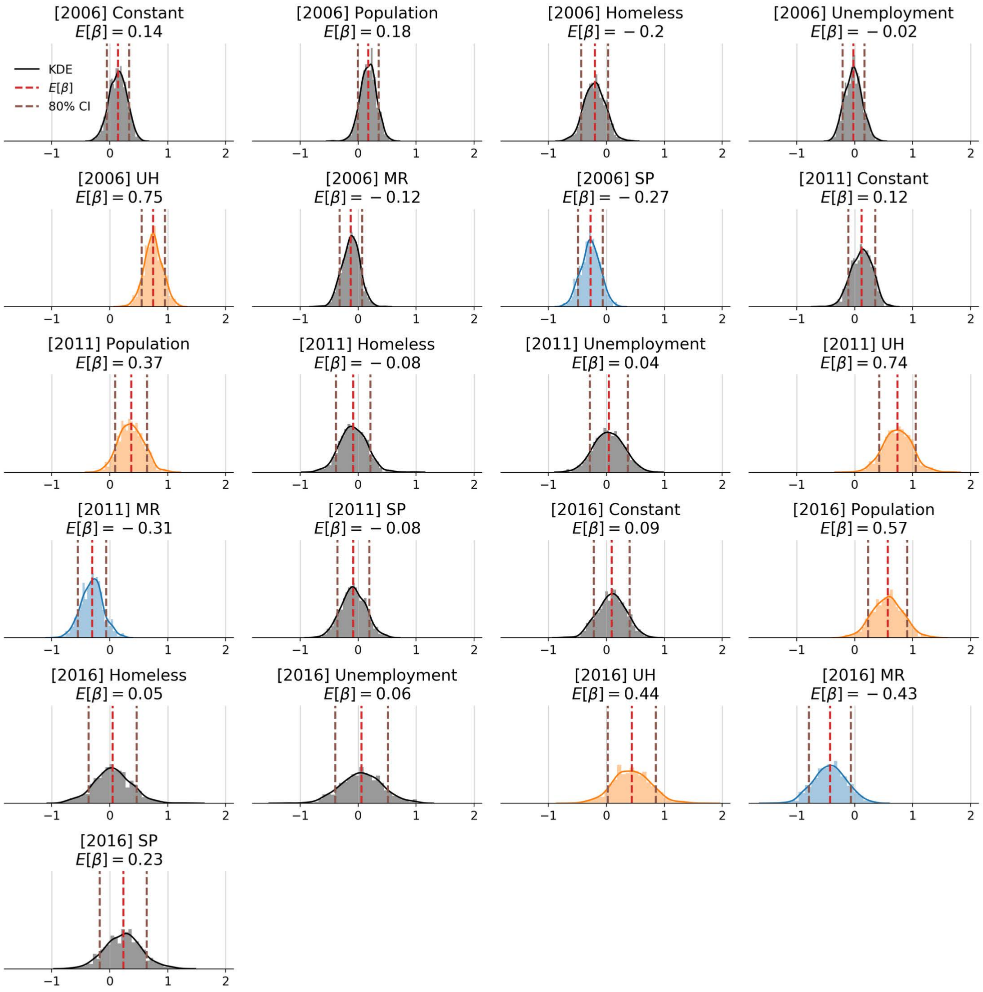

S8 Fig. Distributions of β for the weighted spatial model. We show the kernel density estimation of β coefficient for each feature in the design tensor. Distributions that are significantly above the mean are colored in orange while distributions significantly below the mean are colored in blue as measured by the 80% CI.

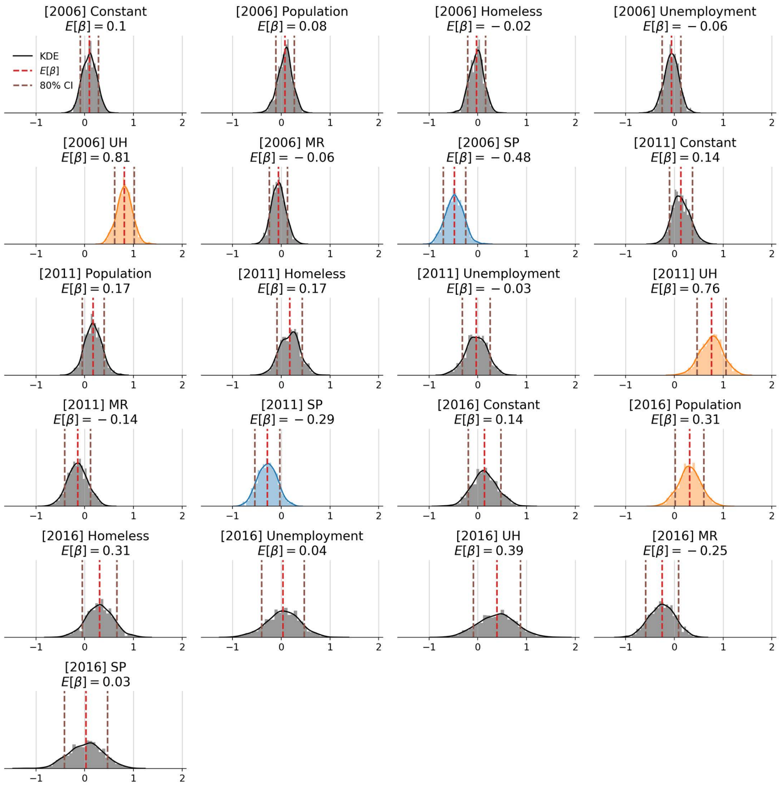

S9 Fig. Distributions of γ for the weighted spatial model. We show the kernel density estimation of γ coefficient for each feature in the design tensor. Distributions that are significantly above the mean are colored in orange while distributions significantly below the mean are colored in blue as measured by the 80% CI.

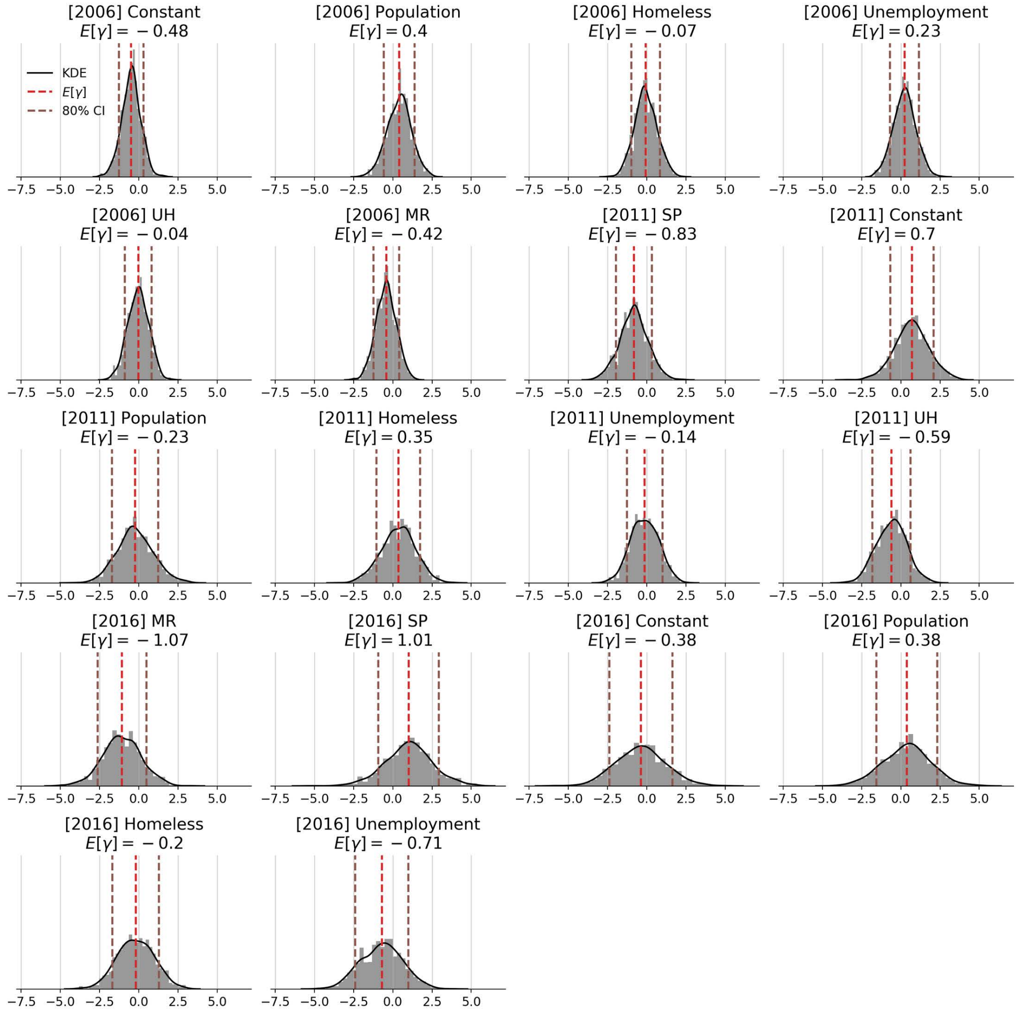

S10 Fig. Ego networks of each district demonstrating sociospatial factors of mortality for the 2016 baseline model. We display ego networks of each district in Hong Kong and its nearest neighbors in the road and bridge network. The central node (highlighted with a grey box) of each network corresponds to the labelled district. Neighbors are not arranged around the ego district geographically. Node color corresponds to normalized mortality rate and edge color corresponds to signed prediction error for the 2016 WSP model. These ego networks encode a qualitative measure of the sociospatial factors in mortality modeling.

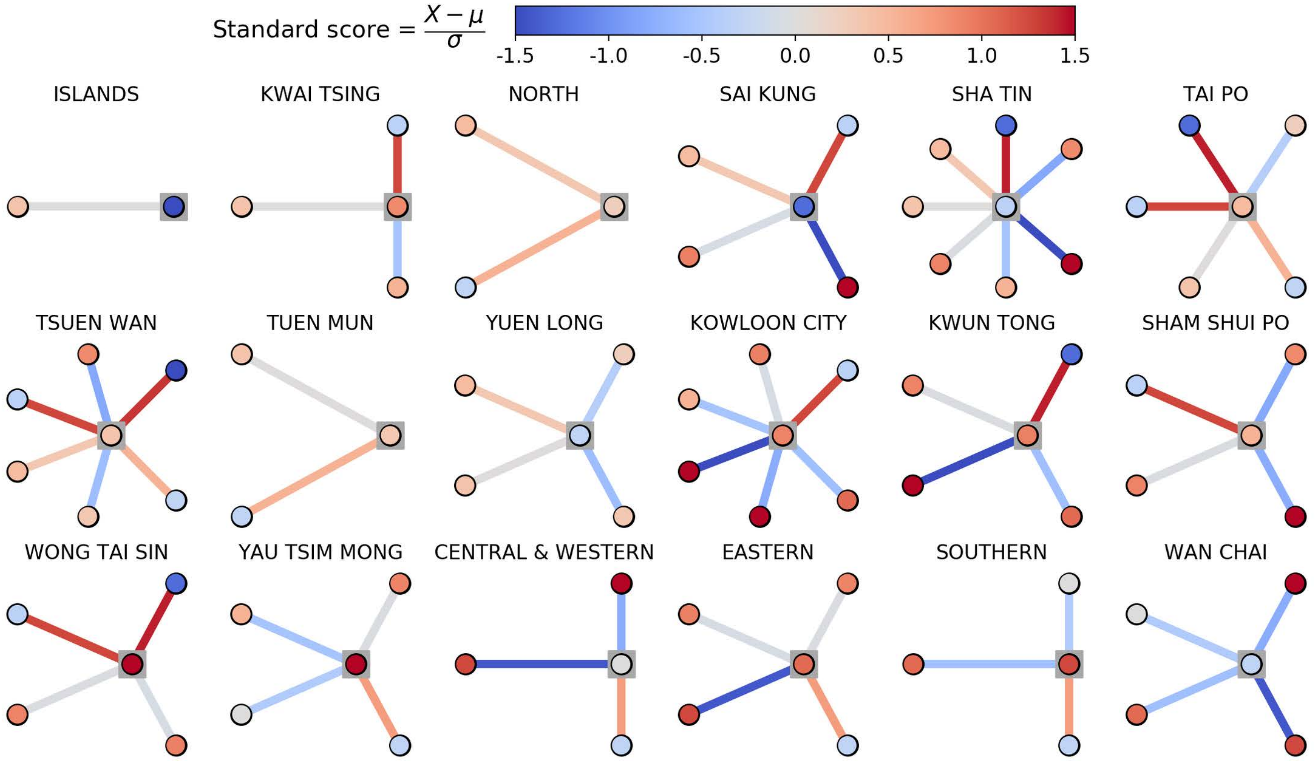

S11 Fig. Ego networks of each district demonstrating sociospatial factors of mortality for the 2016 spatial model. We display ego networks of each district in Hong Kong and its nearest neighbors in the road and bridge network. The central node (highlighted with a grey box) of each network corresponds to the labelled district. Neighbors are not arranged around the ego district geographically. Node color corresponds to normalized mortality rate and edge color corresponds to signed prediction error for the 2016 WSP model. These ego networks encode a qualitative measure of the sociospatial factors in mortality modeling.

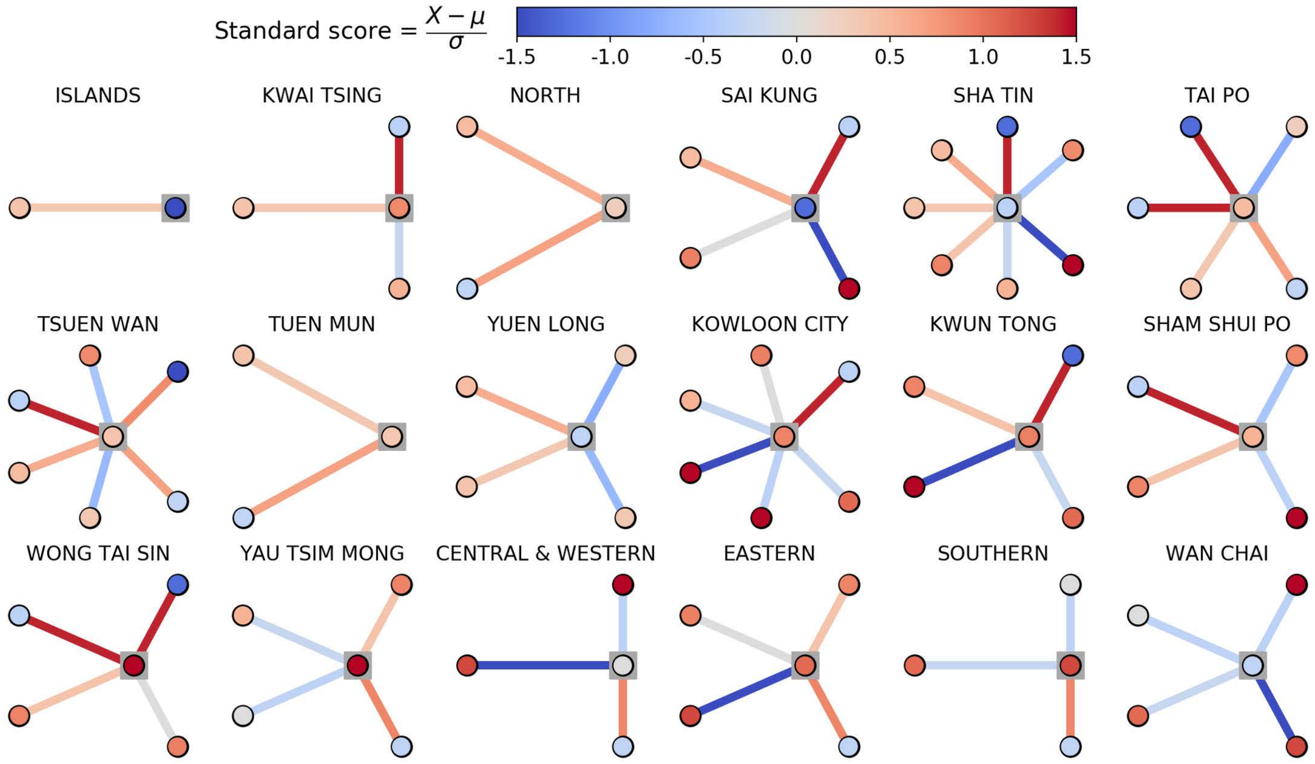

Supplement: S1 File — (PDF) [file pone.0247795.s001.pdf]
